# How to measure and monitor albuminuria in healthy toddlers?

**DOI:** 10.1371/journal.pone.0199309

**Published:** 2018-06-21

**Authors:** Sophie Marielle van den Belt, Valentina Gracchi, Dick de Zeeuw, Hiddo Jan Lambers Heerspink

**Affiliations:** 1 Department of Clinical Pharmacy and Pharmacology, University of Groningen, University Medical Center Groningen, Groningen, The Netherlands; 2 Department of Pediatrics, University of Groningen, University Medical Center Groningen, Groningen, The Netherlands; The University of Tokyo, JAPAN

## Abstract

**Objective:**

Urinary albumin:creatinine ratio (U_ACR_) in first morning void (FMV) urine samples collected over three days is the recommended method for measuring and monitoring albuminuria in adults in the clinical setting. Such a guideline is not available for toddlers and young children. We tested several urine collection strategies for albuminuria measurement in toddlers in a prospective observational study.

**Main outcomes measures:**

Both a FMV and a random daytime urine sample were collected on three consecutive days at week 0, 4, and 8 in toddlers aged 12–48 months. Intra-individual coefficients of variation (CV) of urinary albumin (U_AC_) and U_ACR_ were compared using only the first measurement and using all three measurements per time point. In addition, these were compared with published CV of adults.

**Results:**

A total of 80 toddlers (mean age 26.6 months, 53% male) were included. Intra-individual CV of FMV samples appeared lower than with random samples. The intra-individual CV in U_AC_ or U_ACR_ was smaller using multiple compared to single samples. The lowest intra-individual CV was observed when U_AC_ was measured in FMV over three consecutive days (38.3%). CV of U_AC_ was similar to values published for adults. However, U_ACR_ CV was considerably higher in toddlers.

**Conclusions:**

These data show that—in analogy with adult data—multiple first morning void urine samples should be preferred to single or random urine samples for establishing and monitoring albuminuria in toddlers. Further studies are needed to investigate why creatinine correction for differences in urine dilution is less effective in children.

## Introduction

Measurement of urine albumin is important, both in clinical practice and in epidemiological studies and clinical trials. To accurately quantify albuminuria in the adult population, a 24-hour urine collection is considered the gold standard. This method is rather cumbersome and has thus been replaced in clinical practice by alternatives such as collection of a first morning void (FMV) or a random daytime urine sample. To correct for urine concentration in these samples, albumin concentration is divided by creatinine concentration, where the latter is assumed to be excreted at a constant rate over 24 hours.[1]

To validate these alternative albuminuria measurements against the 24-hour urine albuminuria measurement, several studies have been performed.[2,3] These studies showed that intra-individual variability in U_ACR_, represented by the coefficient of variation (CV), is lower in FMV than in random samples. Furthermore, FMV performs at least as good as a 24-hour urine collection, so that a first morning void urine sample constitutes a good alternative to a 24-hour urine collection.[2]

However, there is still no consensus on how to optimally measure and monitor albuminuria over time in toddlers. Because a 24-hour urine collection is even more cumbersome in young children than in adults and is not feasible for regular controls, it is very important to have reliable alternatives that can be easily implemented in clinical practice.

Therefore, in this study we have compared various urine collection techniques to measure and monitor albuminuria in healthy toddlers. The results are compared with previously reported intra-individual CV’s of albuminuria and creatinine measurements in adults.[2]

## Materials and methods

### Study population

In this observational prospective cohort study, healthy toddlers (12–48 months of age) were recruited from May 2015 until October 2016 via well-baby clinics and children’s daycare centers in Groningen, Drenthe and Friesland (three Northern provinces in the Netherlands). Children with previously diagnosed kidney disease were excluded from the study. Both parents or the legal guardian of the child signed for informed consent. The study was approved by the medical ethical committee of the University Medical Center of Groningen (UMCG) (METc reference number 2014–512) and was conducted according the principles of the declaration of Helsinki.

### Data collection and measurements

**Fig 1:**
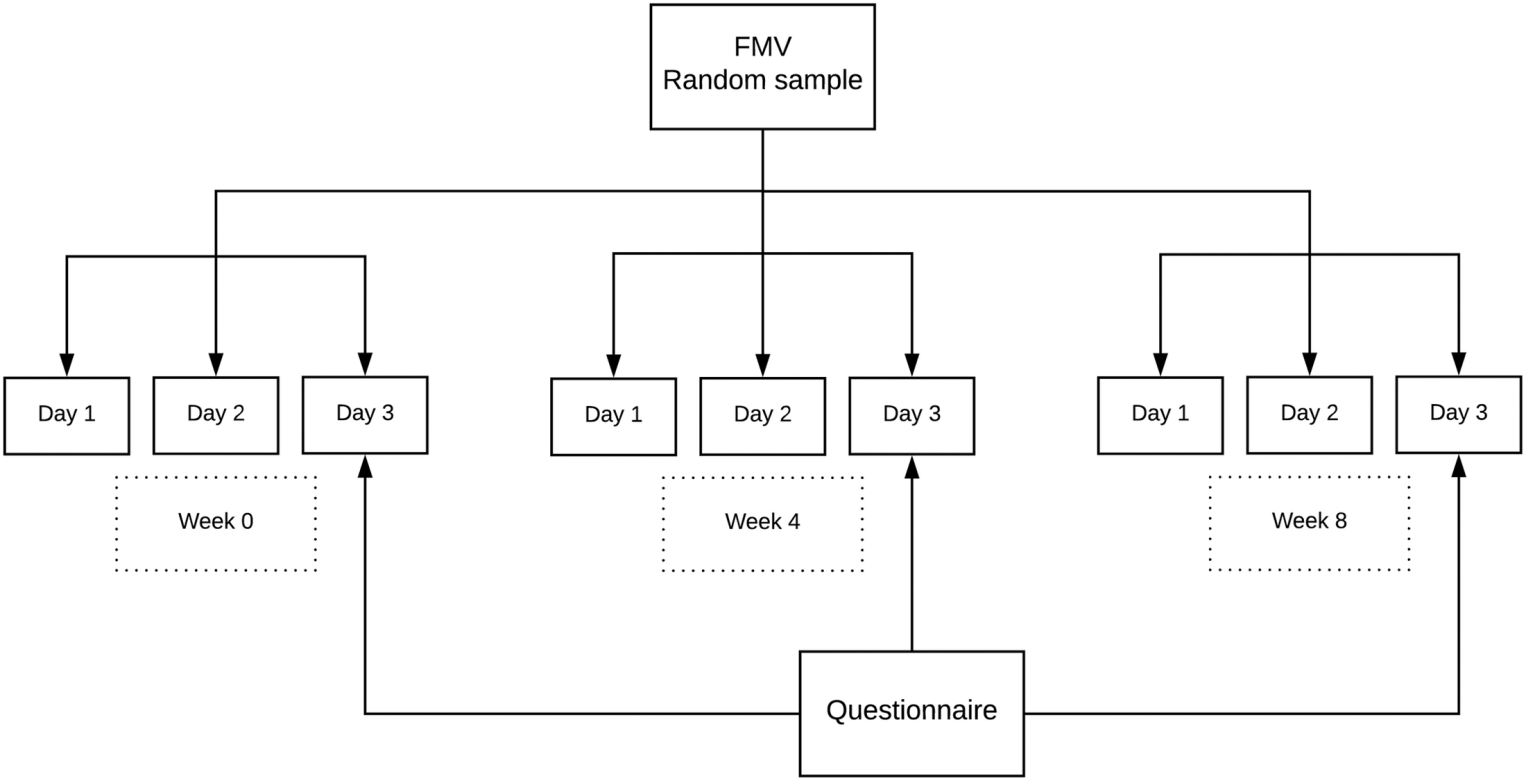
Schedule of data collection.

Data was collected on three consecutive days at three different time points: 0, 4 and 8 weeks. Data consisted of three first morning voids (FMV), three random daytime samples and a questionnaire at each time point (Fig 1). All were collected at home, and sent by mail to the central laboratory of the UMCG. The questionnaire included items regarding date and time of urine collection, possible recent febrile episodes of the child, anthropometric data of child and parents, prenatal and postnatal factors, and information on diabetes, hypertension and cardiovascular disease in parents. To avoid analyzing samples with transient spurious high albuminuria during febrile episodes, urine samples were discarded if the child had fever during the collection period. This occurred eight times.

**Fig 2:**
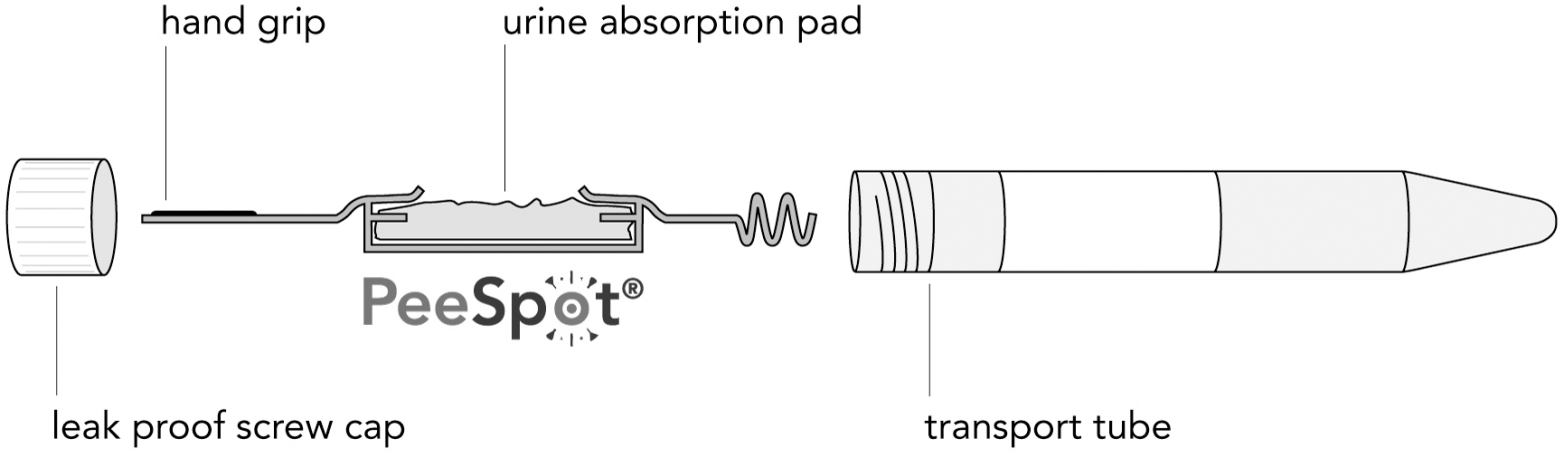
The PeeSpot system.

Urine was collected with the PeeSpot^®^ device, which is a validated tool for urine collection in both children who are continent for urine and children who are not.[4] It consists of a urine absorption pad, a holder, a tube and a lid (Fig 2). In children who are not continent yet, the absorption pad can be placed in the diaper and removed after micturition. Continent children can void over the absorption pad while placed in the holder. After voiding, the pad and holder are placed in the tube, closed with the lid and kept in the refrigerator until sending to the laboratory. After completing the urine collections, the PeeSpots were placed in a safety bag and sent to the central laboratory in an envelope for biological materials (PolyMed, DaklaPack Europe) together with the questionnaire. Upon arrival at the laboratory, samples were processed within 24 hours. Previous studies have shown that albuminuria remains stable during storage on room temperature up to seven days.[4,5] Tubes were centrifuged for 5 minutes on 350G (Rotina 35R). Urinary albumin concentration (U_AC_) was measured with the Roche Modular P using the immunoturbimetric assay and expressed in mg/L. Urinary creatinine concentration (U_CR_) was measured with the Roche Modular P using creatinase to sarcosine oxidase based colorimetric method and expressed in g/L. To correct U_AC_ for urine dilution, urinary albumin:creatinine ratio (U_ACR_) was assessed. Urine samples were tested for leukocytes with urinary dipsticks (Combur-test^®^ strips), in order to exclude an intercurrent urinary tract infection.

### Sample size calculation

Before starting the study, we calculated that a study population of 100 subjects would provide 80% power in detecting a CV between 14% and 24% (difference of 5% relative to 19%), assuming a SD of the within subject CV of 20%. However, in an interim analysis halfway the study, we discovered that the SD of the within subject CV was considerably lower (17.5%). Therefore, we decided to include 80 participants.

### Statistical analyses

Descriptive characteristics of the study population were reported as mean ± standard deviation. U_AC_ and U_ACR_ are represented by geometric mean and sample standard deviation. Geometric mean was used as albuminuria has a non-parametric distribution. In order to assess the intra-individual variability of albuminuria, individual standard deviation and individual geometric mean were assessed to calculate coefficients of variation (CV) of albuminuria per individual with the following formula: $\frac{standard\;deviation}{geometric\;mean}*100\%.$ Coefficients of variation were calculated using a single measurement (first day) per time point and using all three measurements per time point. The prevalence of microalbuminuria was assessed using the definitions that are currently advocated in the paediatric literature (U_AC_ ≥ 20 mg/L or U_ACR_ ≥ 30 mg/g).[6] Differences between CV’s were tested using Wilcoxon matched-pairs signed-ranks test. A two-sided P-value of ≤0.05 was considered to indicate statistical significance. Data were analyzed using STATA version 13.1. Raw data are available in S1 File.

## Results

Of the 95 children of whom informed consent was signed, a total of 80 toddlers (mean age 26.6 months (SD 10.8), 53% male) completed follow-up and were included in this analysis. The remaining 15 children were lost to follow-up. The main characteristics of the study population are shown in Table 1.

**Table 1:** Characteristics of the study population (n = 80).

**Child**	
Age, months (SD)	26.6 (10.8)
Male, n (%)	42 (53)
Body weight, kg (SD)	13.6 (3.0)
Height, cm (SD)	91.4 (9.7)
BMI, kg/m2 (SD)	16.2 (1.5)
Albuminuria at first time point; - U_AC_, mg/L (2.5–97.5^th^ percentile) - U_ACR_, mg/g (2.5–97.5^th^ percentile) - U_CR_ (2.5–97.5^th^ percentile)	5.1 (3.0–43.1) 16.3 (4.2–153.0) 3.5 (0.3–9.4)
Microalbuminuria^#^ at first time point (FMV) - U_AC_, % - U_ACR_, %	5.2% 28.6%
**Antenatal and postnatal factors**	
Birth weight, g (SD)	3518 (545)
Smoking during pregnancy, n (%)	9 (11.3)
Gestational diabetes, n (%)	3 (3.8)
Gestational hypertension, n (%)	3 (3.8)
Breast feeding, n (%)	68 (85)
**Parental: mother**	
Diabetes, n (%)	4 (5)
Hypertension, n (%)	1 (1)
Heart disease, n (%)	0 (0)
**Parental: father**	
Diabetes, n (%)	1 (1)
Hypertension, n (%)	0 (0)
Heart disease, n (%)	0 (0)

^#^Microalbuminuria defined as U_AC_ ≥ 20 mg/L or U_ACR_ ≥ 30 mg/g.

### Variability in albuminuria

Table 2 shows that FMV collections provide lower CV’s in albuminuria (both for U_AC_ and U_ACR_) than random sample collections, although the differences did not always reach statistical significance. In addition, Table 2 shows that three samples per time point show lower CV’s in albuminuria (both U_AC_ and U_ACR_) compared to a single collection per time point. The lowest median intra-individual CV was observed when U_AC_ was measured in FMV on three consecutive days (38.3%). When comparing the CV’s of albumin concentration with albumin:creatinine ratio, U_ACR_ consistently showed a higher CV. This was the case both with a single urine sample per time point or three urine samples per time point and with either FMV or random samples.

**Table 2:** Intra-individual coefficients of variation by number of samples and by collection time (median (25^th^– 75^th^ percentile).

	Single urine sample per time point			Three urine samples per time point				
	FMV	Random	P-value^1^	FMV	Random	P-value^2^	P-value^3^	P-value^4^
**U**_**AC**_	45.8% (17.5–99.8)	44.7% (11.8–85.6)	0.94	38.3% (22.1–56.7)	41.7% (23.4–64.1))	0.66	0.01	0.16
**U**_**ACR**_	54.3% (26.9–114.6)	81.6% (39.5–144.3)	0.05	44.5% (27.4–74.2)	61.7% (34.1–90.4)	0.10	0.06	0.002
**U**_**CR**_	42.3% (20.4–77.6)	58.4% (35.6–87.7)	0.03	27.8% (15.8–57.3)	46.4% (23.9–69.5)	0.03	0.001	<0.001

^1^FMV single sample per time period vs random single sample per time period

^2^FMV three samples per time period vs random three samples per time period

^3^FMV single sample per time period vs FMV three samples per time period

^4^Random single sample per time period vs random three samples per time period

#### Impact of albuminuria measurement on prevalence of microalbuminuria

In clinical practice, urine albumin excretion is classified in normo-, micro, and macroalbuminuria,[6] or recently by normal to mildly increased, moderately increased and severely increased.[7] In adults, microalbuminuria is diagnosed when at least two out of three FMV samples have values in the microalbuminuric range. We calculated the prevalence of microalbuminuria using this adult guideline (at least two of three first samples per time point U_AC_ ≥ 20mg/l or U_ACR_ ≥ 30mg/mg) as well as using the geometric mean of the three consecutive samples (geometric mean of 3 consecutive samples within time point U_AC_ ≥ 20mg/l or U_ACR_ ≥ 30mg/mg). It is clear from the results in Table 3 that microalbuminuria prevalence (based on U_AC_) is very low (around 2%) using the adult guideline, and is higher (around 5%) using the geometric mean. Interestingly, creatinine correction gives very high prevalences (17.5 to 33.8%) when using both the adult guideline and the geometric mean.

**Table 3:** Prevalence of microalbuminuria.

		“Adult—Guideline” microalbuminuria^1^	“Geometric Mean” Microalbuminuria^2^		
			Week 0	Week 4	Week 8
**U**_**AC**_	**FMV**	2.5%	5.2%	5.6%	4.3%
	**Random sample**	1.3%	6.4%	0%	8.5%
**U**_**ACR**_	**FMV**	21.3%	28.6%	28.2%	28.6%
	**Random sample**	17.5%	33.3%	33.8%	33.3%

^1^ defined as at least two of three first samples per time point U_AC_ ≥ 20mg/l or U_ACR_ ≥ 30mg/mg

^2^ defined as geometric mean of 3 consecutive samples within time point U_AC_ ≥ 20mg/l or U_ACR_ ≥ 30mg/mg

### Comparison with intra-individual coefficients of variation in adults

We compared the intra-individual CV found in this study with results from a previous study in adults.[2] In this study 241 healthy adults were included and a FMV and random sample were collected a week 0, week 3 and week 6. Table 4 shows that intra-individual CV in U_AC_ and U_CR_ in this population were similar. However, U_ACR_ CV in our cohort was much higher.

**Table 4:** Intra-individual coefficients of variation of albuminuria in adults (Witte et al. JASN 2009) based on three urine collections.

	FMV	Random
**U**_**AC**_	30.9% (19.4–47.8)	40.9% (27.8–70.2)
**U**_**ACR**_	19.1% (11.6–28.4)	35.8 (17.6–55.6)
**U**_**CR**_	23.5% (13.8–66.7)	31.3% (19.1–56.1)

## Discussion

In this study we compared for the first time various urine collection strategies to optimally measure and monitor albuminuria in toddlers. As expected, albuminuria CV’s are lower when measured in first morning void compared to random samples, and when done with 3 consecutive urine collections versus one collection. This supports implementation of multiple first morning void urine samples for establishing and monitoring albuminuria in toddlers, conform to adult guidelines.

However, we also identified differences with the adult population. In adults, urine albumin correction for urine creatinine (urine albumin:creatinine ratio) helps to correct for differences in urine dilution and reduces variability in the measurements in first morning void or random urine samples. In adults this indeed leads to a more precise assessment of albuminuria and a smaller intra-individual variability in albuminuria. Surprisingly, in our study we observed higher CV’s when U_ACR_ was used as compared to U_AC_. This could be due to a larger variability of urine creatinine in children. However, we did not observe a difference in urinary creatinine CV’s between adults and toddlers. We therefore do not have a clear explanation why creatinine correction does not work in toddlers, other than that urine creatinine measurement itself is flawed in children.

Another difference we encountered with the adult population is that when using urine albumin correction for creatinine in children, the prevalence of microalbuminuria (defined as U_ACR_ ≥ 30mg/mg) also increases substantially as compared to the prevalence defined on the basis of U_AC_ (≥ 20mg/l). The most likely explanation for this is the fact that children have a lower muscle mass, which accounts for an overall lower urinary creatinine excretion and therefore higher physiologic values of U_ACR_. Indeed, multiple studies reported that U_ACR_ is higher in younger children.[8,9] To solve these issues, a large cohort study in different age groups should be performed to investigate differences in U_AC_ and U_ACR_ in FMV samples and compare these results with 24-hour urine albumin excretion to establish optimal age-dependent reference values for U_ACR_. In addition, the urine creatinine measurement itself in children should be validated and compared with adults to find an explanation why U_ACR_ correction does not work in children. At this stage, we recommend to interpret U_ACR_ in young children with extreme caution.

A few studies have reported the prevalence of microalbuminuria in children in the general population.[8–12] Most of these studies used single urine samples collected at a single time point.[8,9,11,12] However, none of these studies have systematically compared the within person variability of albuminuria in healthy toddlers. This is important since the within person variation in albuminuria over time may impact on the accuracy of epidemiological data and the monitoring of albuminuria.

This study has several limitations. The most important is that the albumin measured in the urine samples could not be compared to the gold standard in assessing albuminuria, i.e. 24-hour urine collection. Unfortunately, a 24-hour urine collection in incontinent children is only possible by placing a urinary catheter. In our opinion, this intervention would not have is ethically acceptable for a preliminary study in healthy children, the population we were interested in. Furthermore, by comparing two strategies of urine collection that can be easily performed by the parents at home, we intended to search for both the most reliable and the most practical method for assessing and monitoring albuminuria. A second limitation is that all the urine sampling has taken place at home, so there was no control on whether the collections were performed in the right way and no control on clinical condition of the child. However, by recording date, time of collections, information about clinical condition of the child, possible febrile episodes, and by testing for urinary leucocytes, we intended to screen for factors that could bias the results. Moreover, also in clinical practice most urine collections will be performed at home, especially when FMV samples are required, so our study protocol does mimic clinical situations.

In conclusion, in this study we have compared clinical feasible strategies for establishing and monitoring albuminuria in toddlers. Based on our findings, the most optimal strategy is to measure U_AC_ in FMV samples on three consecutive days, and to repeat measurements in time. Unlike in adults, urine creatinine correction seems not to improve the accuracy of the measurement. Further studies into the creatinine measurement itself as well as into the influence of muscle mass on the use of urine creatinine corrections, are needed to standardize the albuminuria measurements and definitions between children and adults.

## Supporting information

S1 File Raw data. Data used for calculation of the results.
(CSV)
